# Is the urinary neutrophil gelatinase-associated lipocalin concentration in children and adolescents with type 1 diabetes mellitus different from that in healthy children?

**DOI:** 10.11613/BM.2024.020709

**Published:** 2024-06-15

**Authors:** Bernardica Valent Morić, Ivan Šamija, Lavinia La Grasta Sabolić, Adriana Unić, Marijana Miler

**Affiliations:** 1 Department of Pediatrics, Sestre milosrdnice University Hospital Center, Zagreb, Croatia; 2 Catholic University of Croatia, School of Medicine, Zagreb, Croatia; 3 Department of Oncology and Nuclear Medicine, Sestre milosrdnice University Hospital Center, Zagreb, Croatia; 4 Department of Clinical Chemistry, Sestre milosrdnice University Hospital Center, Zagreb, Croatia

**Keywords:** urinary neutrophil gelatinase-associated lipocalin (uNGAL), type 1 diabetes mellitus, albuminuria, diabetic kidney disease, children

## Abstract

**Introduction:**

Diabetic kidney disease (DKD) is one of the major microvascular complications of type 1 diabetes mellitus (T1DM). Some studies suggest that changes of renal tubular components emerge before the glomerular lesions thus introducing the concept of diabetic tubulopathy with urinary neutrophil gelatinase-associated lipocalin (uNGAL) as a potential marker of DKD. This concept was not confirmed in all studies.

**Materials and methods:**

In 198 T1DM patients with median age 15 years and diabetes duration over one year, an albumin/creatinine ratio (ACR) was determined and uNGAL measured in spot urine sample. Urine samples for ACR and uNGAL were also collected in the control group of 100 healthy children of similar age.

**Results:**

There was no significant difference in uNGAL concentration or uNGAL/creatinine between T1DM children and healthy subjects (6.9 (2.8-20.1) ng/mL *vs* 7.9 (2.9-21.0) ng/mL, P = 0.969 and 6.8 (2.2-18.4) ng/mg *vs* 6.5 (1.9-13.4) ng/mg, P = 0.448, respectively) or between T1DM subjects with albuminuria A2 and albuminuria A1 (P = 0.573 and 0.595, respectively). Among T1DM patients 168 (85%) had normal uNGAL concentrations, while in 30 (15%) patients uNGAL was above the defined cut-off value of 30.9 ng/mL. There was no difference in BMI, HbA1c and diabetes duration between patients with elevated uNGAL compared to those with normal uNGAL.

**Conclusions:**

We found no significant difference in uNGAL concentration or uNGAL/creatinine between T1DM children and healthy subjects or between albuminuria A2 and albuminuria A1 T1DM subjects. Therefore, uNGAL should not be recommended as a single marker for detecting diabetic kidney disease in children and adolescents.

## Graphical abstract

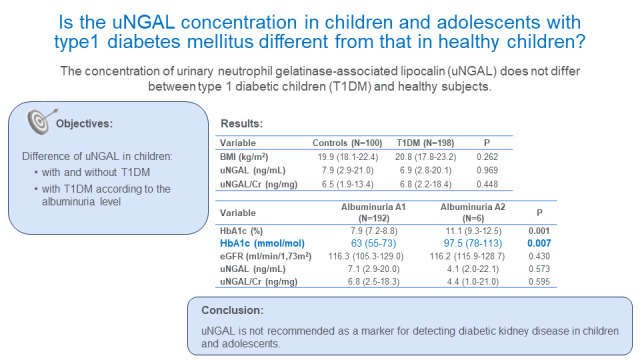

## Highlights

• No significant difference in urinary neutrophil gelatinase-associated lipocalin between type 1 diabetic children and healthy subjects was found

• There was no correlation between urinary neutrophil gelatinase-associated lipocalin and albuminuria

• uNGAL is not recommended as a marker for detecting diabetic kidney disease in children and adolescents

## Introduction

In children the most common form of diabetes is type 1 diabetes mellitus (T1DM). In the Western world it contributes up to 90% of pediatric diabetes mellitus patients and its incidence is increasing (*1*).

Diabetic kidney disease (DKD) is one of the major microvascular complications of T1DM. Although it has been diagnosed less frequently during the last decades, the prevalence of DKD seems to be higher in childhood and adolescence than in patients with later onset of T1DM (*2*). Since T1DM patients with kidney disease are at greater risk of morbidity and mortality compared to those without renal complications, its early diagnosis is of utmost importance in improving clinical management.

Albuminuria is still the most frequently used marker of the vascular complications of T1DM, including DKD (*3*). In the last two decades, it has been shown that histopathological changes of renal tubular and interstitial components emerge before the glomerular lesions and onset of albuminuria A2 thus introducing the concept of diabetic tubulopathy (*4*). To date, various tubular markers have been assessed in the early detection of DKD. Urinary neutrophil gelatinase-associated lipocalin (uNGAL), a marker of proximal tubular injury, has initially been established as a biomarker of acute kidney injury (*5*). Further studies, however, reported the potential role of uNGAL as a marker of chronic kidney disease, including DKD, which precedes the manifestation of typical glomerular lesions (*6*, *7*). In most studies, uNGAL positively correlated to albuminuria levels in both diabetes and pre-diabetes patients (*8*, *9*).

Some studies, however, doubted the accuracy of uNGAL as an individual biomarker of DKD because of the high percentage of false negative subjects in a group of patients with established DKD (*10*). There are also studies in which some T1DM pediatric patients with albuminuria A2 had low uNGAL despite the disease duration from 5 to 6 years (*11*). Furthermore, some investigators concluded that serum and uNGAL do not offer additional prognostic information compared to already established kidney biomarkers (*12*).

We hypothesized that patients with T1DM will have higher concentration of uNGAL compared to healthy children and adolescents and that uNGAL concentration will be higher in T1DM subjects with albuminuria A2 compared to those with albuminuria A1.

Therefore, the aim of our study was to assess the values of uNGAL in T1DM children and adolescents according to the level of albuminuria and to compare clinical and laboratory variables between T1DM patients with different uNGAL categories.

## Materials and methods

### Subjects

This cross-sectional single center study included T1DM children and adolescents aged 5-20 years with diabetes duration over one year. None of the patients were in a diabetes honeymoon period. The study was conducted from January 2017 to May 2018. All patients were followed up at the Department of Pediatrics, Sestre milosrdnice University Hospital Center, Zagreb, Croatia. This investigation was conducted on the same set of T1DM patients as our previous study in which the characteristics of ambulatory blood pressure and its association to albuminuria were examined (*13*). Although these two studies derived from the same experiment, the first study was designed as clinical research with focus on ambulatory blood pressure parameters in T1DM children, while the objective of this study was in a field of clinical chemistry. Furthermore, this investigation has been expanded with the inclusion of a control group.

The study also included 100 healthy controls who attended the Department of Pediatrics for minor health problems (headache, abdominal or chest pain without proven organic disease). The inclusion criteria for the control group were the age 5-20 years and absence of proven disease. The exclusion criteria for both groups were the use of medications that could affect albuminuria or uNGAL (antihypertensive drugs, steroids, estrogens, nonsteroidal anti-inflammatory drugs) and the presence of leukocyturia which could affect uNGAL.

Informed consent for all subjects in the study as well as from the parents of those under 18 years was obtained. Ethical approval was given by the Ethics Committee of Sestre milosrdnice University Hospital Center, Zagreb, Croatia (approval number: 4433/15-19).

### Methods

Body height and weight were recorded and body mass index (BMI) was calculated in both T1DM and control subjects. Body weight was measured to the nearest 0.1 kg on a standard balance scale with subjects wearing light clothing. Standing height was measured to the nearest 0.5 cm using a standardized fixed stadiometer. Body mass index was expressed as kg/m^2^ and BMI *z*-score for age and sex was calculated for each subject using Kromeyer-Hauschild reference system (*14*).

Pubertal development was estimated according to the Tanner stages where Tanner 1 denotes the prepubertal stage and Tanner 2-5 pubertal stages (*15*, *16*).

In the control group only spot urine samples were collected. In the T1DM group laboratory testing also included glycosylated hemoglobin (HbA1c), serum creatinine and urinalysis.

Two tubes were collected for the determination of serum creatinine and HbA1c concentrations: one tube with clot activator for serum (4.5 mL) and one tube with K_3_EDTA anticoagulant (3 mL) for HbA1c (both Vacuette, Greiner Bio-One GmBH, Kremsmünster, Austria). Serum samples and whole blood for the HbA1c analysis were collected the morning after fasting.

Serum creatinine concentration was measured by an enzymatic method in children under 18 years of age, and by Jaffé’s alkaline picrate method in older children immediately after spontaneous clotting for at least 10 minutes and centrifugation of the sample at 4141xg for 7 minutes. Glycosylated hemoglobin was measured using an enzymatic method immediately after preparation of the hemolysate. Both measurements were performed according to the manufacturer’s original instructions using the Architect c8000 (Abbott, Abbott Park, IL, USA).

The creatinine concentration in urine was measured immediately after sampling in 10 mL urine tubes without additive (Vacuette, Greiner Bio-One GmBH, Kremsmünster, Austria). The concentration was measured using the Jaffé alkaline picrate method according to the manufacturer’s original instructions on the Architect c8000 (Abbott, Abbott Park, IL, USA).

Urine spot samples for uNGAL were stored frozen at - 60 °C and analyzed at the end of the inclusion period. Concentrations of uNGAL was measured using commercially available chemiluminescent microparticle immunoassay on Abbott Architect i2000SR analyzer (Abbott Laboratories, Illinois, USA). All assays were conducted in accordance with the manufacturer’s instructions.

To categorize subjects into normal or elevated uNGAL group, we used a cut-off value of 30.9 ng/mL for the NGAL concentration in urine. This value was determined according to the study (not published) in our laboratory on healthy children. Values above this cut-off were considered elevated.

According to the manufacturer’s specifications, the limit of quantification (LoQ) for NGAL is 10 ng/mL, with a limit of blank (LoB) between 0.1 and 0.6 ng/mL and a limit of detection (LoD) between 0.7 and 1.0 ng/mL. Our method verification confirmed the manufacturer’s stated coefficient of variation (CV) of 10%. Intra-assay repeatability and inter-assay precision were measured and yielded CVs of 2.27%, 1.59% and 2.04% for control samples with concentrations of 20 ng/mL, 200 ng/mL and 1200 ng/mL, respectively. For the same samples, the inter-assay precision CVs were 2.63%, 2.13% and 1.88%.

For T1DM subjects urine albumin and creatinine were analyzed from the first morning sample on three occasions at three months intervals starting from the point of inclusion for each patient. According to the International Society for Pediatric and Adolescent Diabetes (ISPAD) 2018 Guidelines the patients were classified as albuminuria A2 group if a minimum of two out of three samples had albumin/creatinine ratio (ACR) 2.5-25 mg/mmol (22.1-221.2 mg/g) in males and 3.5-25 mg/mmol (30.9-221.2 mg/g) in females (*17*). Values under the lower limit of albuminuria A2 (< 2.5 mg/mmol or < 22.1 mg/g in males and < 3.5 mg/mmol or < 30.9 mg/g in females) denoted albuminuria A1 while values above the upper limit of albuminuria A2 (> 25 mg/mmol or > 221.2 mg/g, in both males and females) denoted albuminuria A3. According to the level of albuminuria, T1DM children were categorized as albuminuria A1 or albuminuria A2 group. Urinary albumin was measured by the immunonephelometric method (Behring Nephelometer Analyzer II, Siemens, Malvern, USA). For calculation of estimated glomerular filtration rate for those up to 18 years the Schwartz formula was used, while for adolescents over 18 years 2021 CKD-EPI Creatinine formula was used (www.mdcalc.com/calc/3939/ckd-epi-equations-glomerular-filtration-rate-gfr) (*18*, *19*).

### Statistical analysis

The normality of data distribution was tested using Kolmogorov-Smirnov test. Comparison between groups was performed using the Mann-Whitney U-test for not normally distributed numerical variables, and the Chi-square test for nominal variables. MedCalc version 11.5.1.0 (MedCalc Software, Mariakerke, Belgium) was used for the analysis. Kruskal-Wallis test was used to calculate differences between more than 2 groups of patients with Student-Newman-Keuls *post-hoc* analysis. Data are presented as medians and interquartile range, or median with a range from minimum to maximum for age. Categorical data are presented as percentages or proportions. P value of ≤ 0.05 was considered statistically significant.

## Results

The study included 198 T1DM children and adolescents (109 girls and 89 boys). Their clinical and metabolic characteristics are shown in Table 1. The median age of our T1DM patients was 15 (5-20) years with a median diabetes duration of 7 (3-10) years. Our control group was slightly younger compared to the T1DM group without differences in anthropometric characteristics. There was no difference in BMI between T1DM and the control group as well as between T1DM subjects with albuminuria A1 and albuminuria A2.

**Table 1:** The differences in clinical and metabolic characteristics of the control subjects and T1DM subjects subdivided by the categories of albuminuria

**Variable**	**Controls** **(N=100)**	**Type I diabetic children (total)** **(N=198)**	**P**	**Type I diabetic children with** **albuminuria A1** **(N=192)**	**Type I diabetic children with** **albuminuria A2** **(N=6)**	**P**
Age (years)	14 (6-18)	15 (5-20)	0.004	15 (5-20)	16 (5-18)	0.425
Sex (m, proportion)	0.44	0.45	0.981	0.45	1.0	1.000
BMI (kg/m^2^)	19.9 (18.1-22.4)	20.8 (17.8-23.2)	0.262	20.8 (17.8-23.3)	21.2 (20.1-22.3)	0.651
BMI SDS	0.38 (-0.38-0.93)	0.39 (- 0.17-1.00)	0.623	0.39 (- 0.17-1.00)	0.29 (- 0.38-0.60)	0.691
Diabetes duration (years)	/	6 (3-10)	/	6 (3-9)	8 (7-11)	0.334
HbA1c (%) HbA1c (mmol/mol)	/	7.9 (7.2-8.8) 63 (55-73)	/	7.9 (7.2-8.8) 63 (55-73)	11.1 (9.3-12.5) 97.5 (78-113)	0.001 0.007
eGFR (mL/min/1.73m^2^)	/	116 (105-129)	/	116 (105-129)	116 (116-129)	0.430
Data are presented as medians and interquartile range, or median with a range from minimum to maximum for age. Categorical data are presented as percentages or proportions. T1DM - type 1 diabetes. BMI - body mass index. eGFR - estimated glomerular filtration rate. HbA1c - hemoglobin A1c. SDS - standard deviation score. P value of ≤ 0.05 was considered statistically significant.						

Of 198 T1DM subjects, 192 (97%) had albuminuria A1 while only 6 (3%) had albuminuria A2. None of the subjects had albuminuria A3. Children with albuminuria A2 had significantly higher HbA1c compared to T1DM subjects with albuminuria A1 with median values 11.1% or 98 mmol/mol (9.3-12.5% or 78-113 mmol/mol) *vs* 7.9% or 63 mmol/mol (7.2-8.8% or 55-73 mmol/mol), respectively (P = 0.001). There was no difference in anthropometric characteristics, disease duration or glomerular filtration rate between these two groups.

We found no difference in uNGAL and uNGAL/creatinine ratio between T1DM children and control subjects (6.9 (2.8-20.1) ng/mL *vs* 7.9 (2.9-21.0) ng/mL, P = 0.969 and 6.8 (2.2-18.4) ng/mg *vs* 6.5 (1.9-13.4) ng/mg, P = 0.448, respectively) (Table 2). No difference was also found for uNGAL and uNGAL/creatinine ratio between T1DM subjects with albuminuria A1 and albuminuria A2 (P = 0.573 and 0.595, respectively).

**Table 2:** Urinary NGAL and urinary NGAL/creatinine in study participants

**Variable**	**Controls** **(N = 100)**	**Type I diabetic children (total)** **(N = 198)**	**P**	**Type I diabetic children with albuminuria A1** **(N = 192)**	**Type I diabetic children with** **albuminuria A2** **(N = 6)**	**P**
uNGAL (ng/mL)	7.9 (2.9-21.0)	6.9 (2.8-20.1)	0.969	7.1 (2.9-20.0)	4.1 (2.0-22.1)	0.573
uNGAL/Cr (ng/mg)	6.5 (1.9-13.4)	6.8 (2.2-18.4)	0.448	6.8 (2.5-18.3)	4.4 (1.0-21.0)	0.595
Data are presented as median and interquartile range. uNGAL - urinary neutrophil gelatinase-associated lipocalin. Cr – creatinine. P value of ≤ 0.05 was considered statistically significant.						

Urinary NGAL concentrations were statistically significantly lower in the prepubertal children (Tanner 1) than in the pubertal stages (Tanner 2-5) in both T1DM children and healthy controls (P = 0.001). However, when comparing subgroups by pubertal stage, there were no statistically significant differences in NGAL concentration between the control and T1DM patient groups or between albuminuria types (data not shown). Moreover, eGFR showed no differences according to Tanner stages (P = 0.126). Median concentrations of uNGAL for all prepubertal and pubertal children and adolescents were below the cut-off values (3.1 ng/mL and 9.3 ng/mL, respectively).

Among T1DM patients 168 (84.8%) had normal uNGAL while in 30 (15.2%) patients uNGAL was above the defined cut-off value. There were no differences in BMI, HbA1c and diabetes duration between patients with elevated uNGAL compared to those with normal uNGAL (Table 3).

**Table 3:** Characteristics of diabetic patients with normal and elevated urinary NGAL

**Variable**	**Normal uNGAL (≤ 30.9 ng/mL)** **(N = 168)**	**Elevated uNGAL (> 30.9 ng/mL)** **(N = 30)**	**P**
Age (years)	15 (12-18)	16 (14-19)	0.087
Gender (m, proportion)	0.51	0.13	< 0.001
BMI (kg/m^2^)	20.6 (17.4-23.1)	21.3 (19.7-24.9)	0.130
BMI SDS	0.35 (- 0.18-0.91)	0.49 (0.03-1.28)	0.374
Diabetes duration (year)	6 (3-9)	8 (5-11)	0.045
HbA1c (%)	7.9 (7.1-8.8)	8.3 (7.4-9.2)	0.083
HbA1c (mmol/mol)	63 (54-73)	67 (57-77)	0.083
eGFR (mL/min/1.73m^2^)	116 (106-129)	116 (104-128)	0.909
Data are presented as medians and interquartile range, or median with a range from minimum to maximum for age. Categorical data are presented as proportions. BMI - body mass index. uNGAL - urinary neutrophil gelatinase-associated lipocalin. SDS - standard deviation score. HbA1c - hemoglobin A1c. eGFR - estimated glomerular filtration rate. P value of ≤ 0.05 was considered statistically significant.			

## Discussion

The results of our study showed no significant difference in uNGAL or uNGAL/creatinine between T1DM children and healthy subjects or between T1DM subjects with albuminuria A2 and albuminuria A1. Furthermore, we found no association between uNGAL or uNGAL/creatinine with the level of albuminuria. Therefore, our hypothesis was not confirmed.

Contrary to our results, some previous studies showed increased uNGAL concentrations in T1DM children with albuminuria A1 before reduction in glomerular filtration rate compared to nondiabetic control subjects pointing out that these results may indicate early diabetic kidney injury (*20*). Similar results were also observed by Zachwieja *et al.,* who analyzed spot uNGAL in a small group of normoalbuminuric diabetic children and found that it was significantly higher compared to controls (*7*). In our study, uNGAL and uNGAL/creatinine values did not differ between albuminuria A2 and albuminuria A1 T1DM children and adolescents.

At this point, two important aspects of uNGAL analysis should be emphasized. First, it is the lack of standardized reference values for uNGAL in chronic kidney disease, especially in pediatric patients and second is the existence of two analytical methods (ELISA and chemiluminescent platform) which complicates the comparison of results between different studies. Currently, two studies providing a pediatric reference range for uNGAL are available: the chemiluminescent platform reference values published by Cangemi *et al.,* and ELISA reference values published by Bennett *et al.* (*21*, *22*). Although both studies are of good quality including over 300 healthy children, their results differ in some important aspects. The ELISA method demonstrated significant gender differences with higher concentration of uNGAL in females along with differences across the age range with rising concentrations from early childhood to adolescence. On the other hand, the chemiluminescent method showed no significant correlation between uNGAL and both age and gender.

It is obvious that cut-off values for different determination methods (ELISA or chemiluminescent platform) and different age groups can vary considerably. Each manufacturer points out in the instructions for use that each laboratory should check the cut-off values and define its own reference ranges. We performed the study (not published) in our laboratory on healthy children and determined cut-off values of 30.9 ng/mL for the NGAL concentration in urine. Therefore, we used these values in our study as the manufacturer’s values were declared for adults.

Similar to our results, the study of Demir *et al.*, in which, as in our study, the chemiluminescent microparticle immunoassay method was used, showed no statistically significant difference for uNGAL between T1DM and control group, although the difference was significant for uNGAL/creatinine (*9*). Furthermore, the uNGAL in both groups was around mean values for healthy children, significantly lower than the 97th percentile according to the previously mentioned reference values. The authors commented that better glycemic control would explain the lack of elevation in uNGAL concentration which might be also the case in our investigation. In the same study, uNGAL concentration were found to be correlated with ACR as was the case in some other studies (*7*, *23*). We did not confirm such a correlation which is in accordance with some other studies in which no relationship was found between not only for uNGAL and ACR but also for other diabetes indices (diabetes duration, HbA1c concentration) (*20*).

As in some other studies, our investigations also showed no difference in the level of metabolic control between T1DM patients with normal and elevated uNGAL (*7*, *24*). We also found no difference in diabetes duration between the two uNGAL groups and these results were similar to some other studies (*23*).

The possible limitation of our study is the lack of uNGAL follow-up since the dynamics of tubular injury markers might give better insight in kidney injury than just a single urine sample.

In our experience, the lack of significant difference in uNGAL or uNGAL/creatinine between T1DM children and healthy subjects or between T1DM subjects with albuminuria A2 and albuminuria A1 leads to a conclusion that uNGAL should not be recommended as a single marker for detecting diabetic kidney disease in children and adolescents.

## Data Availability

The data generated and analyzed in the presented study are available from the corresponding author on request.
